# Layer-dependent quantum cooperation of electron and hole states in the anomalous semimetal WTe_2_

**DOI:** 10.1038/ncomms10847

**Published:** 2016-02-29

**Authors:** Pranab Kumar Das, D. Di Sante, I. Vobornik, J. Fujii, T. Okuda, E. Bruyer, A. Gyenis, B. E. Feldman, J. Tao, R. Ciancio, G. Rossi, M. N. Ali, S. Picozzi, A. Yadzani, G. Panaccione, R. J. Cava

**Affiliations:** 1 Istituto Officina dei Materiali (IOM)-CNR, Laboratorio TASC, in Area Science Park, S.S.14, Km 163.5, I-34149 Trieste, Italy; 2 International Centre for Theoretical Physics (ICTP), Strada Costiera 11, I-34100 Trieste, Italy; 3 Consiglio Nazionale delle Ricerche—CNR-SPIN, I-67100 L'Aquila, Italy; 4 Department of Physical and Chemical Sciences, University of L'Aquila, Via Vetoio, I-67100 L'Aquila, Italy; 5 Hiroshima Synchrotron Radiation Center (HSRC), Hiroshima University, 2-313 Kagamiyama, Higashi-Hiroshima 739-0046, Japan; 6 Joseph Henry Laboratories and Department of Physics, Princeton University, Princeton, New Jersey 08544, USA; 7 Department of Condensed Matter Physics and Materials Science, Brookhaven National Laboratory, Upton, New York 11973, USA; 8 Dipartimento di Fisica, Università di Milano, Via Celoria 16, I-20133 Milano, Italy; 9 Department of Chemistry, Princeton University, Princeton, New Jersey 08544, USA

## Abstract

The behaviour of electrons and holes in a crystal lattice is a fundamental quantum phenomenon, accounting for a rich variety of material properties. Boosted by the remarkable electronic and physical properties of two-dimensional materials such as graphene and topological insulators, transition metal dichalcogenides have recently received renewed attention. In this context, the anomalous bulk properties of semimetallic WTe_2_ have attracted considerable interest. Here we report angle- and spin-resolved photoemission spectroscopy of WTe_2_ single crystals, through which we disentangle the role of W and Te atoms in the formation of the band structure and identify the interplay of charge, spin and orbital degrees of freedom. Supported by first-principles calculations and high-resolution surface topography, we reveal the existence of a layer-dependent behaviour. The balance of electron and hole states is found only when considering at least three Te–W–Te layers, showing that the behaviour of WTe_2_ is not strictly two dimensional.

## Abstract (web summary)

Tungsten ditelluride is a semi-metallic two-dimensional material that has exhibited large magnetoresistance. Here, the authors use angle- and spin-resolved photoemission spectroscopy to investigate the band structure of this transition metal dichalcogenide and identify layer-dependent electronic behaviour.

## Graphical abstract

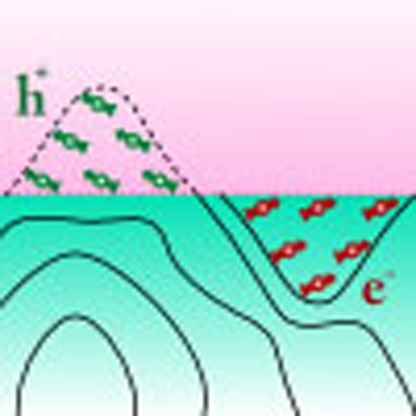

Transition metal dichalcogenides (TMDs) are a group of layered materials with chemical formula MX_2_, where M is a transition metal and X can be S, Se or Te. Their properties span from pure insulators to good metals, and they also exhibit various low-temperature phenomena such as metal–insulator transitions, superconductivity and charge density waves^1^. An important aspect of TMDs is the presence of anisotropic bonding with different strengths: the X–M–X building blocks are stacked along the crystallographic *c*-direction, and while the inter-layer interaction is mainly of weak van der Waals type, the intra-layer bonding between the atoms is strong and covalent. Dimensionality is thus expected to play a significant role in TMDs, because the transition from a single or a few layers to bulk implies significant change in the symmetry of the orbitals and in quantum confinement, producing important differences in the electronic structure.

Among TMDs, WTe_2_ is special because it displays an additional structural distortion: the tungsten atoms form zigzag chains along the crystallographic *a* axis, producing a quasi one-dimensional arrangement. Moreover, WTe_2_ is a semimetal with a reduced density of states at the Fermi level coming from a small overlap between valence and conduction bands without a band gap. It exhibits an extremely large uniaxial positive magnetoresistance with no saturation up to a magnetic field as high as 60 T (refs 2), which has been attributed to perfect electron and hole compensation. In addition, the presence of two heavy elements points to the importance of spin–orbit coupling (SOC) in determining the details of the Fermi surface and/or the relevant low-energy excitations of the system.

Here, by combining results of scanning tunnelling microscopy (STM), spin- and angle-resolved photoemission spectroscopy (ARPES), and layer-resolved *ab initio* calculations, we explored the details and the evolution of electron and hole states in WTe_2_. Our data reveal significant differences between surface and bulk electronic properties, with a clear evolution as a function of depth from the surface. The Fermi surface measured by ARPES is significantly reduced with respect to the one calculated for the bulk, indicating the presence of a reduced, yet still balanced, number of electrons and holes at the surface and near-surface region. The importance of SOC is directly shown by spin-resolved ARPES measurements, and the data are consistent with our theoretical calculations.

## Results

### STM measurements to characterize the surface properties

**Figure 1:**
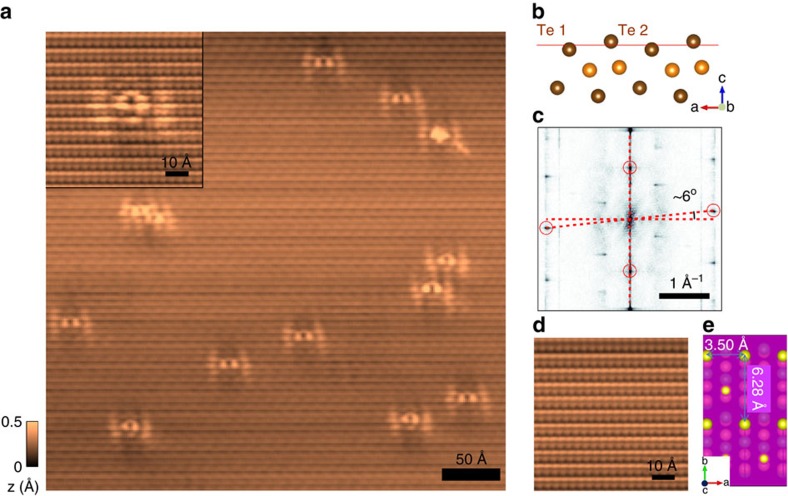
Surface topography and structure of WTe_2_. (**a**) Topographic image of a 400 Å × 400 Å area on the (001) surface of WTe_2_ with several ‘tie-fighter'-like impurities (*V*_bias_=−80 mV, *I*_tunneling_=100 pA and *T*=30 K). Inset: Zoom-in on an individual defect. (**b**) Side schematic view of the WTe_2_ lattice highlighting the two inequivalent Te atoms. (**c**) Fourier transform of the topograph in **a** reveals Bragg peaks corresponding to the atomic corrugation (red circles indicate the first-order Bragg peaks) and the anomalous angle between the crystallographic axes on the surface (6° in this sample). The degree of the angular distortion varied a few degrees between different samples. (**d**) Zoom-in on a clean area displays the surface Te atoms. (**e**) Top schematic view of the WTe_2_ (001) surface highlighting the surface Te atoms and the unit cell dimensions.

To visualize the surface quality of WTe_2_, we performed high-resolution scanning tunnelling microscopy (Fig. 1). The STM images reveal that the top layer is composed of two inequivalent Te atoms, labelled Te1 and Te2 in Fig. 1b (see also Supplementary Note 1, and Supplementary Fig. 1). The surface is atomically ordered with extremely high quality and low impurity concentration, with approximately one underlying defect per 3,000 atoms observed (Fig. 1a), corresponding to ≈9 × 10^11^ defects cm^−2^. In addition, the topographic image and its Fourier transform (Fig. 1c) clearly show that the electronic structure of the surface has an angular distortion, similar to that reported previously^4^,^5^. We observe the distortion at multiple tip-sample biases, in multiple samples, and with different STM tips, indicating that this finding is not related to thermal drift, tip artifacts or a particular energy (see Supplementary Note 2, and Supplementary Figs 2–4). Comparison with TEM data suggest that the distortion is due to surface reconstruction and model calculations for a single WTe_2_ monolayer show that it does not significantly affect the band structure (see Supplementary Note 2, and Supplementary Fig. 5).

### ARPES results and DFT calculations

**Figure 2:**
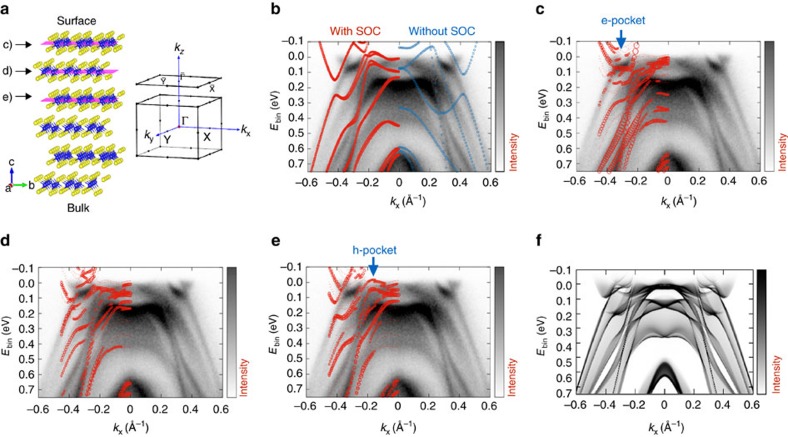
Evolution of band structure with number of layers. (**a**) Crystal structure of WTe_2_ with the bulk and surface Brillouin zones on the right. On the left, the slab model used in the theoretical calculations, consisting of six Te–W–Te layers; each layer is formed by hexagonally packed W sandwiched between two Te layers. (**b**–**e**) ARPES measurements (*hv*=68 eV, *T*=77 K) of the electronic structure along the ΓX high symmetry direction (along the W chains); (**b**) bulk electronic structures as calculated with SOC (red bands at negative momenta) and without SOC (blue bands at positive momenta); (**c**) theoretical bands projected on the topmost WTe_2_ planes; (**d**,**e**) theoretical bands projected on second and third plane, respectively. In **c**,**e**, blue arrows mark the positions of the theoretical electron and hole pockets, respectively. In **b**,**e**, the size of the circles is proportional to the weight of the layer-resolved orbital character, calculated as the sum of the orbital characters of all the atoms belonging to the respective layer. (**f**) The theoretical surface spectral function *A*(**k**,*E*).

We next explore the detailed electronic structure of WTe_2_ and the related layer dependence of the electron and hole states. In Fig. 2b we show the experimental *E* versus *k* ARPES spectra overlaid with bulk theoretical band structures calculated using density functional theory (DFT) with (red bands on the left) and without (blue bands on the right) SOC, along the reciprocal space line XΓX. Following the standard procedure and assuming WTe_2_ as a pure 2D-layered system, calculations are performed with out-of-plane **k**-vector component *k*_z_=0. We observe well-defined electron and hole pockets at the Fermi energy along the tungsten chain direction (ΓX), confirming previous results^6^. However, a detailed comparison of the observations to bulk theoretical band structure as described above presents some inconsistencies. The theoretical position of the electron pocket is significantly further away from the zone centre and its maximum binding energy is larger than the measured one by more than a factor two. The presence of heavy atoms like W and Te suggests that SOC plays an important role in the interpretation of WTe_2_ spectral features. The changes in the theoretical band structure associated with SOC (Fig. 2b, red curves) illustrate that relativistic effects cannot be neglected in WTe_2_, and although we obtain somewhat better results, a quantitative agreement is not found in either case. We also note a non-negligible *k*_z_ band dispersion^2^,^6^ when comparing experimental spectra with theoretical bands for reciprocal lines parallel to ΓX at different *k*_z_ momenta, ranging from the Brillouin zone centre (*k*_z_=0) to the Brillouin zone edge (*k*_z_=*π*/*c*) (see Supplementary Figs 6–9). However, including *k*_z_ dispersion does not result in an improved agreement between calculations and experiment, since none of the calculated band structures for any of the given *k*_z_ values can fully reproduce the measured dispersion (see Supplementary Fig. 6).

We find a significant improvement, however, when using a more realistic model that takes into account the contribution of individual layers (that is, all the bulk *k*_z_ momenta at the same time projected on the surface Brillouin zone). This model is based on a supercell made by van der Waals-bonded WTe_2_ planes stacked along the [001] direction (Fig. 2a). From the large number of bands in the supercell calculations, we are able to isolate the individual contribution of each WTe_2_ plane in the stack by projecting the electronic band structure onto the atoms belonging to that given plane only. We note that the experimental ARPES spectra correspond to a weighted spectral intensity from the different layers probed, and the contributions of deeper layers are exponentially attenuated by the inelastic mean free path of photoelectrons. Superimposing the theoretical bands on the experimental spectra, and projecting onto the first, second and third WTe_2_ planes (Fig. 2c–e, respectively), we filter the layer-dependent information about the electronic structure because we are able to correlate individual features of the layer-resolved calculations in the experimental spectra. In this analysis, the size of the plotted circles is proportional to the contribution of the given WTe_2_ plane: the bigger the circle, the larger the contribution of that plane to the spectral features. We thus observe in Fig. 2c that the electron pocket is located at 0.35 Å^−1^ from the zone centre (highlighted by the blue arrow) and is present even when only the first Te–W–Te layer is considered. The hole pocket (blue arrow in panel e) starts appearing only when taking into account the presence of the third layer, and thus has a more bulk-like character. Furthermore, we cannot exclude the existence of a small hole pocket at Γ, as recently proposed by theoretical findings^7^ for an isolated WTe_2_ monolayer (note that in the same work the electron pockets are closer to the zone centre, similar to our case); a similar hole pocket has also recently been observed in other ARPES experiments^8^. In our case (Fig. 2), the hole pocket is fully occupied and the range of explored temperatures is well below the Lifshitz transition recently reported at above 160 K (ref. 9). Given that the hole pocket is very close to *E*_F_, the difference could be due to temperature induced shift of the chemical potential as proposed in ref. 9. Moreover, hole pockets close to each other on either side of the Γ point would justify the unusual magnetic breakdown observed in quantum oscillations^10^.

We further stress that our slab calculations do not reveal the existence of distinct surface states. This is supported by the comparison of bulk theoretical band structures for different *k*_z_ momenta with the electronic states of the slab (see Supplementary Fig. 7). In fact, all bulk bands lie within the continuum of the slab's bulk band structure projected onto the surface Brillouin zone. Indeed, the bands shown in Fig. 2c–e overlaid to ARPES spectra are bulk states showing substantial spectral weight on the topmost surface layers; this seems in analogy to what is observed in MoS_2_ and many other systems, like iron pnictides, with cleaved neutral surfaces^11^,^12^.

The present analysis not only provides a quantitative agreement between experiment and theory in terms of dispersion and momentum space locations of electronic states, but clearly shows the presence of a layer-dependent evolution of electron and hole states: a bulk-like electronic structure characterized by the electron-hole charge balance is obtained only when more than two Te–W–Te layers are taken into account. Our results clearly display some analogies with other ‘less than 3D' materials: in the case of topological insulators, for example, it has been theoretically predicted and experimentally confirmed not only that the critical thickness of six quintuple layers is needed to set the topological properties of the surface but also that the spin–orbital texture of a topological insulator evolves in a layer-dependent manner, extending over several nanometers from the surface^13^,^14^.

Given that a nearly equal concentration of electrons and holes is a necessary condition for non-saturating magnetoresistance to be observed in two-component systems^15^,^16^, the present observation has important implications towards the realization of devices based on few-layer TMDs^17^.

To further check the robustness and the reliability of our theoretical interpretation, we used two different approaches: (i) an *ab initio* tight-binding model for a 40 layers slab (thickness about 42 nm, see Supplementary Fig. 10); (ii) a renormalization scheme for semi-infinite systems to calculate the surface spectral function, shown in Fig. 2f (ref. 18; see ‘Methods' section for details). These checks aimed at excluding spurious effects arising from the interaction between the two extreme surfaces (always present in slab calculations if the number of layers is not large enough). The theoretical bands of Fig. 2f provide a direct link with the measured *E* versus *k* intensity maps (except for dipole matrix elements, neglected in our calculations). By using both methods, most of the experimental features are well reproduced, in particular the hole and electron pockets near *E*_F_ as well as the bands at higher binding energy. Moreover, no remarkable differences are observed in comparison with the DFT results for the six-layer-thick slab.

**Figure 3:**
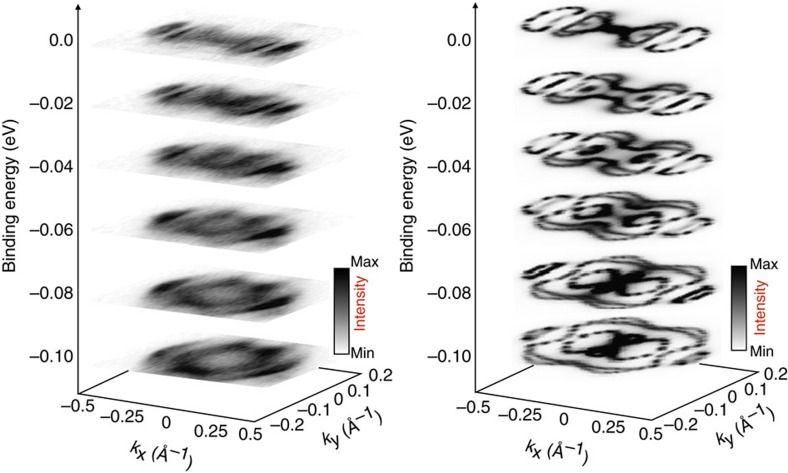
Fermi surface topography. Constant energy cuts from *E*_F_ down to 100 meV binding energy (in steps of 20 meV). Left: experimental spectra measured at a photon energy of 68 eV and at 77 K, right: theoretical calculations for bulk WTe_2_ at *k*_z_=0.

Figure 3 shows the experimental (left panel) and bulk theoretical (right panel) constant energy cuts from *E*_F_ down to 100 meV binding energy, in steps of 20 meV. The theoretical results qualitatively reproduce the main features of the Fermi surface, including the shape and the distribution of the pockets and a finite intensity at Γ (that is, confirming the presence of a possible hole pocket at Γ). However, the calculated bulk features have a significantly larger area than the measured Fermi surface, providing further evidence that simply recasting bulk calculations is insufficient to explain the experimentally observed ARPES features. The area of the Fermi surface calculated by the bulk theoretical model is larger than the area of the observed Fermi surface, which implies that the total number of carriers is larger in the bulk than on the surface. In spite of this difference, the comparison shows that the balance between electrons and holes is maintained in both cases, to within the sensitivity of our technique (see also Supplementary Fig. 9). This, in turn, indicates that the balancing between electrons and holes, that is, their ‘quantum-cooperation' over different layers, is the dominant factor that determines the macroscopic properties of the system such as also the non-saturating magnetoresistance.

### Spin-resolved ARPES results

**Figure 4:**
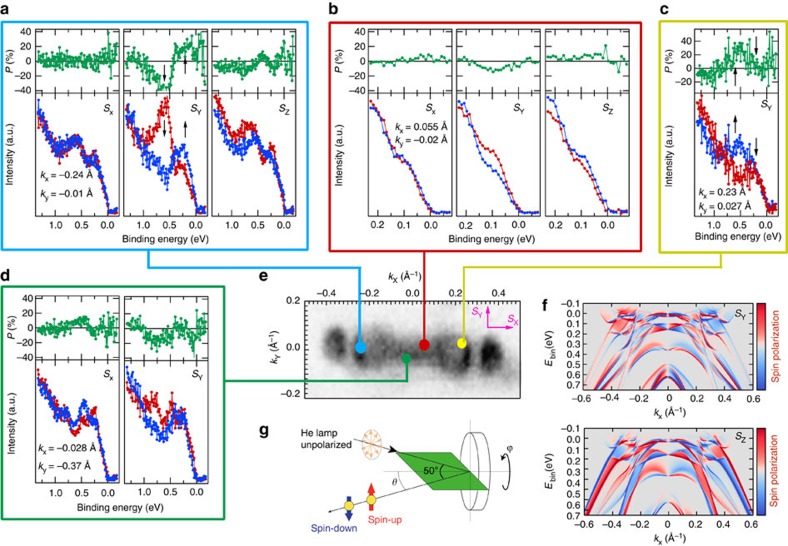
Experimental and theoretical spin polarized band structure of large SOC and non-centro-symmetric WTe_2_. (**a**–**d**) Measured spin-resolved ARPES spectra (red and blue curves) and spin polarization (green curves) at four distinct **k** points as indicated in **e**; (**e**) experimental Fermi surface with the corresponding spin measurement positions; (**f**) calculated band structure along *k*_x_ direction, upper/lower panel shows *S*_y_/*S*_z_ spin orientation. The colour scales represent the degree of spin polarization; red, blue and white refer to up, down and no-spin polarization, respectively; (**g**) experimental geometry of spin-resolved ARPES setup. In **a**, *S*_y_, the down-spin spectrum (red curve) has a larger peak at 0.6 eV (down arrow) and the up-spin spectrum (blue curve) has a larger peak at 0.2 eV (up arrow), whereas at **c**, on the contrary, the up-spin spectrum has a dominating peak at 0.6 eV and the down-spin spectrum has a dominating peak at 0.2 eV, supporting time-reversal effect.

To determine the role of SOC and obtain insight into the spin texture, we performed spin-resolved measurements at a number of **k** points in the Brillouin zone. Spin-resolved ARPES spectra are presented in Fig. 4, as measured at the hole pocket (panels a, c; blue and yellow circles), close to Γ on the ΓX line (panel b, red circle) and close to Γ, but off the ΓX line (panel d, green circle). Panel f shows the calculated spin-resolved band structure along ΓX.

We measure a sizeable spin polarization (*P*) for an extended binding energy range. It reaches more than 35% for *P*_*y*_ at a binding energy of 0.55 eV (panel a, black arrows). This indicates that there must not only be a broken space inversion symmetry but also a significant influence of SOC in WTe_2_. The spin texture is quite complex, with large oscillations in value and sign of *P* in a narrow energy window, clearly visible in both *P*_*y*_ and *P*_*z*_ (Fig. 4a). This observation is confirmed by the calculation in Fig. 4f. Experimental error cannot exclude the presence of a finite spin polarization at *E*_F_, but it is negligible with respect to those observed at higher binding energies. The fine spectral structures in the calculation are not visible in the experimental spin polarization measurements, due to the experimental energy and angular resolution. However, we obtain notable qualitative agreement, as detailed below. The *P*_*x*_ component is zero along the ΓX line (Fig. 4a,b), while we observe non-zero *P*_*x*_ component for points away from ΓX (Fig. 4d). This means that the electron spin along ΓX is perpendicular to the W chains, in agreement with calculations that predict only large *P*_*y*_ and *P*_*z*_ spin polarizations (Fig. 4f). The results shown in Fig. 4a,b (at the hole pocket) and Fig. 4f reveal the presence of both in-plane and out-of-plane components of the spin polarization, in contrast with ordinary Rashba systems where *P* is only in-plane^19^. The spin polarization changes sign upon crossing the Brillouin zone centre, that is, the sign of *P* is reversed at 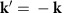, as experimentally confirmed in Fig. 4a,c, which show data taken at positive and negative *k*_*x*_ values. This indicates that time reversal symmetry is preserved, that is, 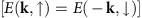 and that the observed spin polarization of bands has nonmagnetic origin. A large spin polarization of electronic bands has been recently reported in the semiconducting TMD WSe_2_, where *P* occurs due to the local asymmetry of layers^20^ (consecutive Se–W–Se layers have opposite net dipole moment, which modulate the spin texture strongly even though the global inversion symmetry is preserved in the crystal). Unlike WSe_2_, the crystal structure of WTe_2_ is non-centrosymmetric. Therefore, by symmetry consideration one naturally expects a lifting of the spin degeneracy via spin–orbit coupling. The amplitude of spin polarization depends on many different factors (that is, orbital character, band gap, electric fields and so on), but primarily on the strength of the SOC; therefore, our results indicate that SOC is clearly reflected in the spectral function of WTe_2_, as also recently reported by CD-ARPES results^8^.

## Discussion

Considering our present results from a more general perspective, it is important to underline that exact carrier compensation is a necessary, yet not sufficient, condition for a non-saturating quadratic behaviour of magnetoresistance^15^. Bismuth provides an example where compensation is observed, but quadratic dependence of resistance in a magnetic field and non-saturation are not^15^,^21^. Other prerequisites for behaviour similar to that observed in WTe_2_ are: (a) low density of impurities and defects and (b) a carrier density far from the quantum limit^22^,^23^. Here we have shown that condition (a) is nearly fulfilled as shown by STM results, and condition (b) is supported by the reported low resistivity and high mobility in WTe_2_ (refs 2, 7, 24). These observations suggest that future experiments exploring the relationship between electronic structure and magnetoresistance in WTe_2_ would be worthwhile and that WTe_2_ could be a potential candidate to form an excitonic dielectric in the Abrikosov sense^22^,^25^.

In summary, our theoretical and experimental ARPES findings provide clear evidence that the electronic properties of WTe_2_ display a layer-dependent evolution from surface to bulk, that is, it cannot be considered a priori as a non-interacting 2D-layered system, in agreement also with recent temperature-dependent magnetoresistance measurements^26^. The balance between the hole and electron states, representing one of the crucial conditions for the non-saturating magnetoresistance in this system, is established only beyond finite number of layers (three) and maintained in the bulk. This consideration provides a fundamental input for future exploitation of TMDs in general, and WTe_2_ in particular, in devices and heterogeneous interfaces.

## Methods

### ARPES experiments

ARPES experiments were performed at APE-IOM beamline^27^ at the ELETTRA Sincrotrone Trieste. High quality surfaces were obtained by cleaving the samples in UHV at a base pressure of 1 × 10^−10^ mbar. The crystallographic orientation was examined by low-energy electron diffraction (LEED) patterns. Core levels, valence-band and Fermi surface measurements were performed using a high-resolution VG-SCIENTA DA30 electron analyzer, in a photon energy range of 20–100 eV, with an angular and energy resolution better than 0.2 deg and 20 meV, respectively. The low temperature data are collected at 16 K using a liquid helium cooled cryostat.

### STM

Before the measurements, the samples were cleaved in ultra-high vacuum at room temperature and immediately transferred to our home-built variable temperature STM. Measurements were performed at 28 K.

### Spin-resolved ARPES

Spin-resolved ARPES experiments were performed at HiSOR^28^. The spin-resolved spectra were measured by means of VLEED spin detector using Fe-O target; two VLEED detectors positioned orthogonally are able to measure the *x*, *y* (in-plane) and *z* (out of plane) components of the spin polarization. Standard He laboratory light source (*hν*=21.22 eV) was used as incident beam at various temperatures down to 10 K. The asymmetry of the spin polarization was quantified by reversing the current through a coil. The analyser resolution of SR-ARPES was 60 meV and the angular resolution was 1.5 degrees. The spin asymmetry *A* is given by *A*= (*I*_+_−*I*_−_)/(*I*_+_+*I*_−_). The actual polarization *P* depends on the instrument and detector setup (target), and is given by *P=A/S*_eff_, where *S*_eff_ is the effective Sherman function value corresponding to the detector and instrumental setup used. Here we have used *S*_eff_=0.2. Next the up and down spin component *S*_↑_, *S*_↓_ can be calculated following the expression 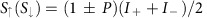.

### Density functional theory calculations

Supercell calculations were performed using the generalized gradient approximation (GGA) as implemented in the DFT code Vienna Ab-Initio Simulation Package (VASP)^29^,^30^. Atomic positions were fully relaxed starting from data in ref. 31. We used the projector augmented wave method by explicitly treating six valence electrons both for W and Te, while *d* electrons of Te were kept within the core of the PBE pseudopotentials. Integration over the first Brillouin zone was made with a 12 × 8 × 1 Monkhorst-Pack k-mesh centred at Γ (24 × 12 × 2 for bulk calculations). For all the simulations, a 400 eV plane-wave energy cut-off was used. Spin–orbit coupling has been self-consistently taken into account. Dipole corrections, as implemented in VASP, were applied along the *z* direction to counteract any spurious electric field that might arise from periodic boundary conditions in the presence of a dipole moment normal to the surface in a slab geometry (with 20 Å-thick layer of vacuum). To model surface as well as more bulk features, we considered a slab containing six planes of WTe_2_ stacked onto each other along the [001] direction. The effects of van der Waals interactions between WTe_2_ planes has been properly taken into account by the DFT-D2 method of Grimme^32^, but no significant differences in the electronic properties have been detected with respect to GGA calculations.

To calculate bulk Fermi surfaces reported in Fig. 3 (right panel), we adopt a two step procedure: (i) first, we projected the bulk Hamiltonian onto a basis made of *s* and *d* W-centered, and *s* and *p* Te-centered orbitals, for a total of 112 Wannier functions, by means of the WANNIER90 package; (ii) subsequently, the Wannier Hamiltonian is used to build up the bulk Green's function as *G*(**k**,*w*)=1/(*w*−*H*(**k**)+*iδ*), which, in turn, gives the Fermi surface maps at the chosen binding energy, *w*. Theoretical spectral functions have been calculated within the framework of the surface renormalization method^18^ based on the same Wannier Hamiltonian as described before. The surface spectral function is defined as *A*(**k**,*w*)=−(1/π)*ImG*_surf_(**k**,*w*), where *G*_surf_(**k**,*w*) represents the angular and energy resolved surface Green's function. Major differences between *A*(**k**,w) and ARPES data, as, for example, line intensities and high-**k** features, possibly rely in the absence of transition matrix elements in the theoretical description.

## Additional information

**How to cite this article:** Das, P. K. *et al.* Layer-dependent quantum cooperation of electron and hole states in the anomalous semimetal WTe_2_. *Nat. Commun.* 7:10847 doi: 10.1038/ncomms10847 (2016).

## Supplementary Material

Supplementary Information Supplementary Figures 1-10, Supplementary Notes 1-2 and Supplementary References.

## References

[b1] Wang Q. H. *et al.* Electronics and optoelectronics of two-dimensional transition metal dichalcogenides. Nat. Nanotechnol. 7, 699–712 (2012). 23132225 10.1038/nnano.2012.193

[b2] Ali M. N. *et al.* Large, non-saturating magnetoresistance in WTe_2_. Nature 514, 205–208 (2014). 25219849 10.1038/nature13763

[b3] Crossley A., Myhra S. & Sofield C. J. STM analysis of WTe_2_ surfaces—correlation with crystal and electronic structures. Surf. Sci. 318, 39–45 (1994).

[b4] Hlaa S. W., Marinković V., Prodana A. & Muševiča I. STM/AFM investigations of β-MoTe2, α-MoTe2 and WTe2. Surf. Sci. 352–354, 105–111 (1996).

[b5] Pletikosić I., Ali M. N., Fedorov A. V., Cava R. J. & Valla T. Electronic structure basis for the extraordinary magnetoresistance in WTe_2_. Phys. Rev. Lett. 113, 216601 (2014). 25479512 10.1103/PhysRevLett.113.216601

[b6] Lv H. Y. *et al.* Perfect charge compensation in WTe2 for the extraordinary magnetoresistance: from bulk to monolayer. Europhys. Lett. 110, 37004 (2015).

[b7] Jiang J. *et al.* Signature of strong spin-orbital coupling in the large nonsaturating magnetoresistance material WTe_2_. Phys. Rev. Lett. 115, 166601 (2015). 26550888 10.1103/PhysRevLett.115.166601

[b8] Wu Y. *et al.* Temperature induced Lifshitz transition in WTe_2_. Phys. Rev. Lett. 115, 166602 (2015). 26550889 10.1103/PhysRevLett.115.166602

[b9] Zhu Z. *et al.* Quantum oscillations, termoelectric coefficients, and the Fermi surface of semimetallic WTe_2_. Phys. Rev. Lett. 114, 176601 (2015). 25978245 10.1103/PhysRevLett.114.176601

[b10] Gehlmann M. *et al.* Quasi 2D electronic states with high spin-polarization in centrosymmetric MoS2 bulk crystals. Preprint at http://arxiv.org/abs/1510.04101 (2015). 10.1038/srep26197 PMC4887890 27245646

[b11] Lankau A. *et al.* Absence of surface states for LiFeAs investigated using density functional calculations. Phys. Rev. B 82, 184518 (2010).

[b12] Zhang Y., He K. & Chang C. Crossover of the three-dimensional topological insulator Bi_2_Se_3_ to the two-dimensional limit. Nat. Phys. 6, 584–588 (2010).

[b13] Zhu Z. H. & Levy G. Rashba spin-splitting control at the surface of the topological insulator Bi_2_Se_3_. Phys. Rev. Lett. 107, 186405 (2011). 22107654 10.1103/PhysRevLett.107.186405

[b14] Alekseev P. S. *et al.* Magnetoresistance in two-component systems. Phys. Rev. Lett. 114, 156601 (2015). 25933326 10.1103/PhysRevLett.114.156601

[b15] Parish M. M. & Littlewood P. B. Non-saturating magnetoresistance in heavily disordered semiconductors. Nature 426, 162–165 (2003). 14614501 10.1038/nature02073

[b16] Wang L. *et al.* Tuning Magnetotransportin a Compensated Semimetal at the Atomic Scale. Preprint at http://arxiv.org/abs/1510.04827 (2015). 10.1038/ncomms9892 PMC4673487 26600289

[b17] Henk J. & Schattke W. A subroutine package for computing Green's functions of relaxed surfaces by the renormalization method. Comput. Phys. Commun. 77, 69–83 (1993).

[b18] Hasan M. Z. & Kane C. L. Colloquium: topological insulators. Rev. Mod. Phys. 82, 3045 (2010).

[b19] Riley J. M. *et al.* Direct observation of spin-polarized bulk bands in an inversion-symmetric semiconductor. Nat. Phys. 10, 835–839 (2014).

[b20] Liang T. *et al.* Evidence for massive bulk Dirac fermions in Pb_1-x_Sn_x_Se from Nernst and thermopower experiments. Nat. Commun. 4, 2696 (2013). 24176908 10.1038/ncomms3696

[b21] Abrikosov A. A. The transformation of a semimetal into an exciton dielectric in a strong magnetic field. Sov. Phys. Usp. 15, 662–663 (1973).

[b22] Fenton E. W. Electrical resistivity of semimetals in the extreme quantum limit. J. Low Temp. Phys. 7, 415–432 (1972).

[b23] Ali M. N. *et al.* Correlation of crystal quality and extreme magnetoresistance of WTe_2_. Europhys. Lett. 110, 67002 (2015).

[b24] Abrikosov A. A. Quantum linear magnetoresistance. Europhys. Lett. 49, 789–793 (2000).

[b25] Thoutam L. R. *et al.* Temperature-dependent three-dimensional anisotropy of the magnetoresistance in WTe_2_. Phys. Rev. Lett. 115, 046602 (2015). 26252701 10.1103/PhysRevLett.115.046602

[b26] Panaccione G. *et al.* Advanced photoelectric effect experiment beamline at Elettra: a surface science laboratory coupled with synchrotron radiation. Rev. Sci. Instrum. 80, 043105 (2009). 19405649 10.1063/1.3119364

[b27] Okuda T. *et al.* Efficient spin resolved spectroscopy observation machine at Hiroshima Synchrotron Radiation Center. Rev. Sci. Instrum. 82, 103302 (2011). 22047286 10.1063/1.3648102

[b28] Kresse G. & Furthmüller J. Efficient iterative schemes for *ab initio* total-energy calculations using a plane-wave basis set. Phys. Rev. B 54, 11169 (1996). 10.1103/physrevb.54.11169 9984901

[b29] Kresse G. & Joubert D. From ultrasoft pseudopotentials to the projector augmented-wave method. Phys. Rev. B 59, 1758 (1999).

[b30] Mar A., Jobic S. & Ibers A. Metal-metal vs tellurium-tellurium bonding in WTe_2_ and its ternary variants TaIrTe_4_ and NbIrTe_4_. J. Am. Chem. Soc. 114, 8963 (1992).

[b31] Grimme S. Semiempirical GGA-type density functional constructed with a long-range dispersion correction. Comp. Chem. 27, 1787 (2006). 10.1002/jcc.20495 16955487

[b32] Mostofi A. A. *et al.* wannier90: a tool for obtaining maximally-localised Wannier functions. Comput. Phys. Commun. 178, 685 (2008).

