# Erratum: Layer-dependent quantum cooperation of electron and hole states in the anomalous semimetal WTe_2_

**DOI:** 10.1038/ncomms11355

**Published:** 2016-04-13

**Authors:** Pranab Kumar Das, D. Di Sante, I. Vobornik, J. Fujii, T. Okuda, E. Bruyer, A. Gyenis, B. E. Feldman, J. Tao, R. Ciancio, G. Rossi, M. N. Ali, S. Picozzi, A. Yadzani, G. Panaccione, R. J. Cava

Nature Communications 7, Article number: 10847 10.1038/ncomms10847 (2016); Published 02292016; Updated 04132016.

This Article contains errors in the numbering of the references in the text.

The third sentence of the second paragraph should only refer to reference 2, not references 2 and 3. The correct sentence is:

‘It exhibits an extremely large uniaxial positive magnetoresistance with no saturation up to a magnetic field as high as 60 T (ref. 2), which has been attributed to perfect electron and hole compensation.'

All subsequent reference numbers in the text refer to the previous paper in the reference list. For example, 4 in the text refers to the third reference in the list, 5 refers to the fourth paper in the list and so on throughout up to and including 32 in the text, which refers to the 31st paper in the reference list.

The 32nd reference in the list should be cited in the first sentence of the final paragraph of the Methods section. The correct sentence is:

‘To calculate bulk Fermi surfaces reported in Fig. 3 (right panel), we adopt a two step procedure: (i) first, we projected the bulk Hamiltonian onto a basis made of *s* and *d* W-centered, and *s* and *p* Te-centered orbitals, for a total of 112 Wannier functions, by means of the WANNIER90 package^32^; (ii) subsequently, the Wannier Hamiltonian is used to build up the bulk Green's function as *G*(**k**,*w*)=1/(*w*−*H*(**k**)+*iδ*), which, in turn, gives the Fermi surface maps at the chosen binding energy, *w*.'

