# Author Correction: Layer-dependent quantum cooperation of electron and hole states in the anomalous semimetal WTe_2_

**DOI:** 10.1038/s41467-019-08649-5

**Published:** 2019-02-06

**Authors:** Pranab Kumar Das, D. Di Sante, I. Vobornik, J. Fujii, T. Okuda, E. Bruyer, A. Gyenis, B. E. Feldman, J. Tao, R. Ciancio, G. Rossi, M. N. Ali, S. Picozzi, A. Yazdani, G. Panaccione, R. J. Cava

**Affiliations:** 1 Istituto Officina dei Materiali (IOM)-CNR, Laboratorio TASC, in Area Science Park, S.S.14, Km 163.5, I-34149 Trieste, Italy; 2 International Centre for Theoretical Physics (ICTP), Strada Costiera 11, I-34100 Trieste, Italy; 3 Consiglio Nazionale delle Ricerche—CNR-SPIN, I-67100 L’Aquila, Italy; 4 Department of Physical and Chemical Sciences, University of L’Aquila, Via Vetoio, I-67100 L’Aquila, Italy; 5 Hiroshima Synchrotron Radiation Center (HSRC), Hiroshima University, 2-313 Kagamiyama, Higashi-Hiroshima, 739-0046 Japan; 6 Joseph Henry Laboratories and Department of Physics, Princeton University, Princeton, New Jersey 08544 USA; 7 Department of Condensed Matter Physics and Materials Science, Brookhaven National Laboratory, Upton, New York 11973 USA; 8 Dipartimento di Fisica, Università di Milano, Via Celoria 16, I-20133 Milano, Italy; 9 Department of Chemistry, Princeton University, Princeton, New Jersey 08544 USA

Correction to: *Nature Communications;* 10.1038/ncomms10847, published online 29 Feb 2016.

This Article contains an error in the spelling of the author A. Yazdani, which is incorrectly given as A. Yadzani. The error has not been fixed in the original PDF and HTML versions of the Article.

