# Supplementary material for: Layer-dependent quantum cooperation of electron and hole states in the anomalous semimetal WTe2
Source: Nat Commun. 2016 Feb 29;7:10847. doi: 10.1038/ncomms10847 (PMC4773464; doi:10.1038/ncomms10847)
Supplement: Supplementary Information — Supplementary Figures 1-10, Supplementary Notes 1-2 and Supplementary References. [file ncomms10847-s1.pdf]

Supplementary Figures

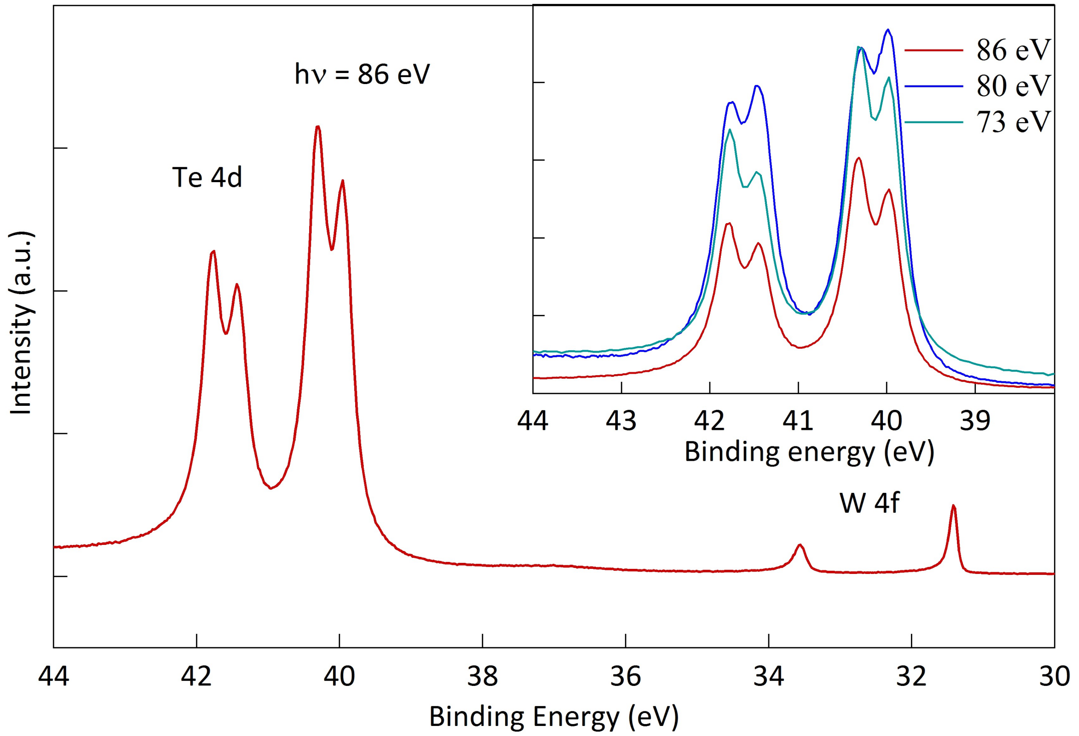

Supplementary Figure 1: Core level spectra. Te 4d and W 4f core level spectra of WTe_2 single crystal, measured at *hv*=86 eV (main panel), while W 4f level are single line peaks, a clear splitting is observed in the Te 4d. In the inset, intensity evolution of the Te 4d core level peak vs. photon energy; the ratio of the split peaks, in each spin orbit partner, changes vs. photon energy as due to photoelectron diffraction effects, thus indicating the presence of two inequivalent Te atoms. The spectra have been measured at temperatures below 77 K.

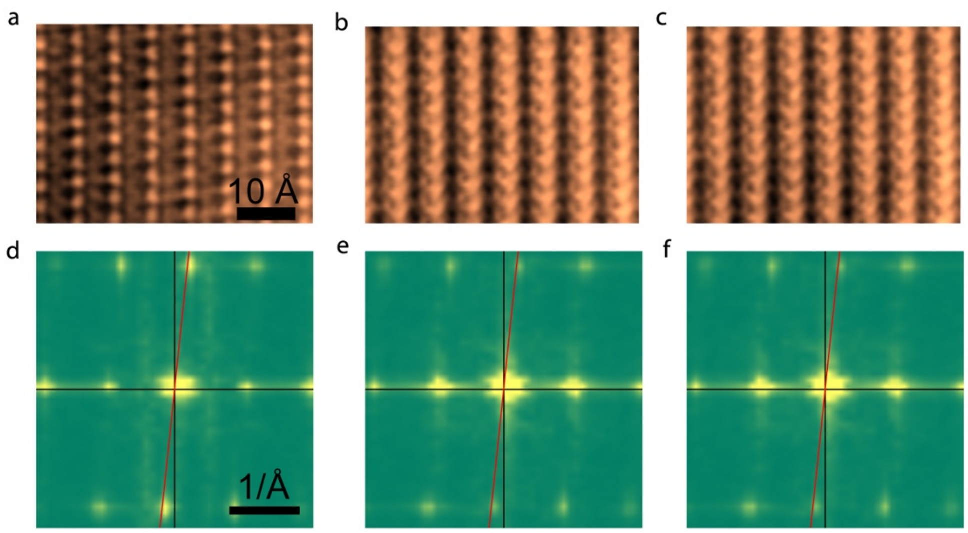

Supplementary Figure 2: Surface distortion study be STM. Panel a-c: Topographic images of the (001) surface of WTe_2 acquired at different bias voltages of 200 mV, 100 mV, -100 mV, respectively, and corresponding Fourier transforms of the topographs (panel d-f), where the surface distortion between the crystallographic axes is found (6° in this sample, independent of tip-sample bias). The degree of the angular distortion varied a few degrees between different samples.

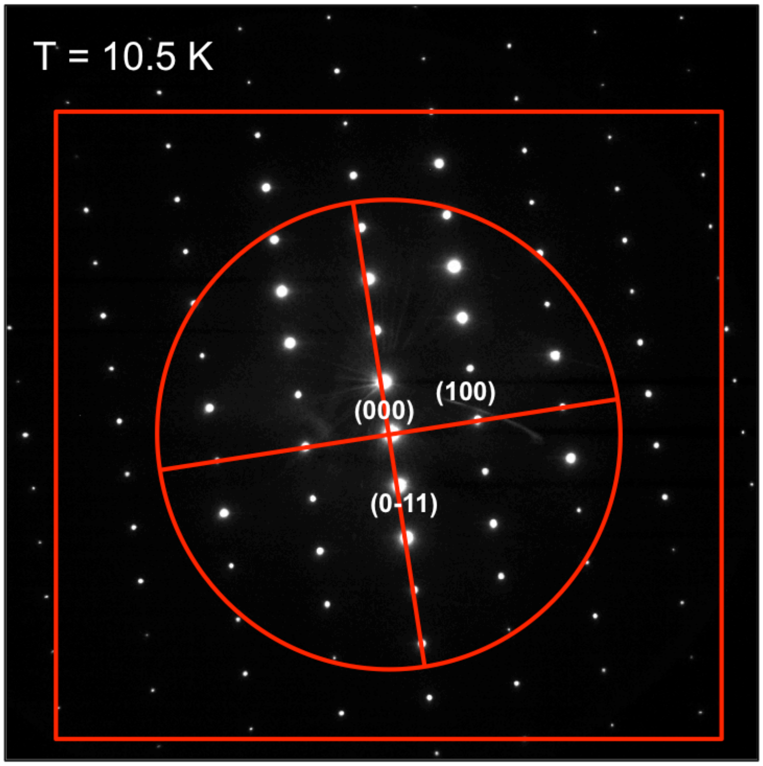

Supplementary Figure 3: TEM measurement. High Resolution Transmission Electron Microscopy of a freshly cleaved WTe_2 crystal.

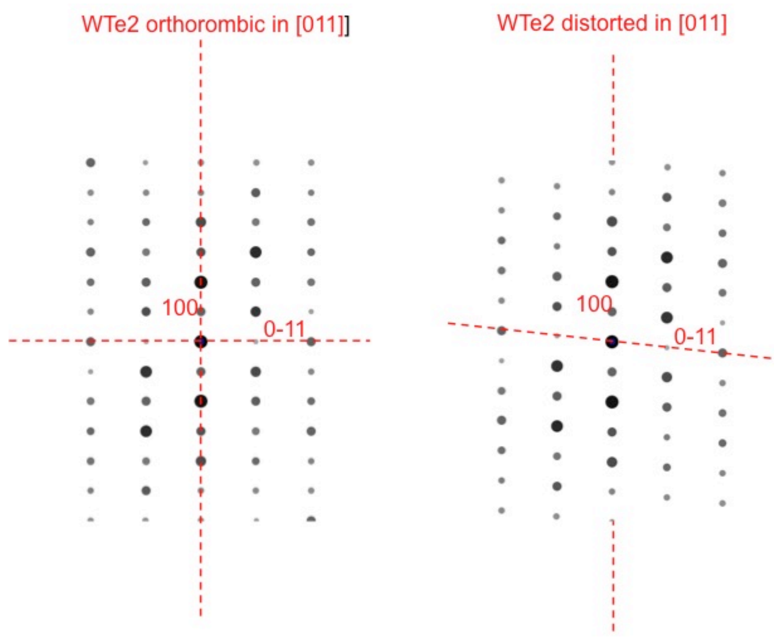

Supplementary Figure 4: Effect of distortion on diffraction pattern. Simulation of non distorted (left) and distorted (right) diffractograms for orthorhombic WTe_2.

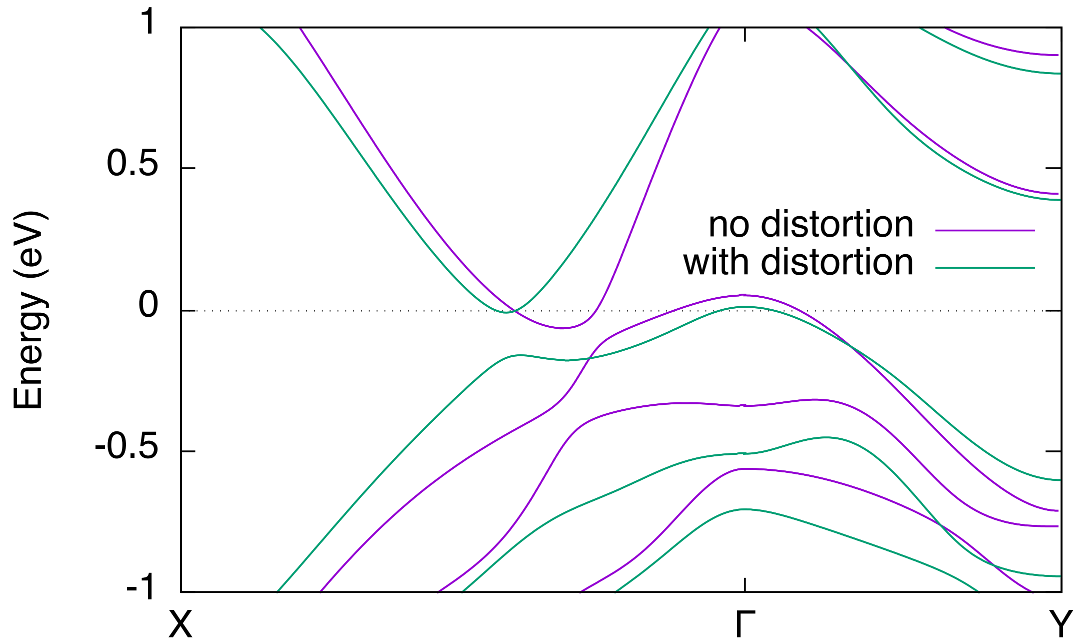

Supplementary Figure 5: Effect of distortion. Band structure calculation of a monolayer of WTe_2 with and without the observed structure distortion of 6 degrees at the surface.

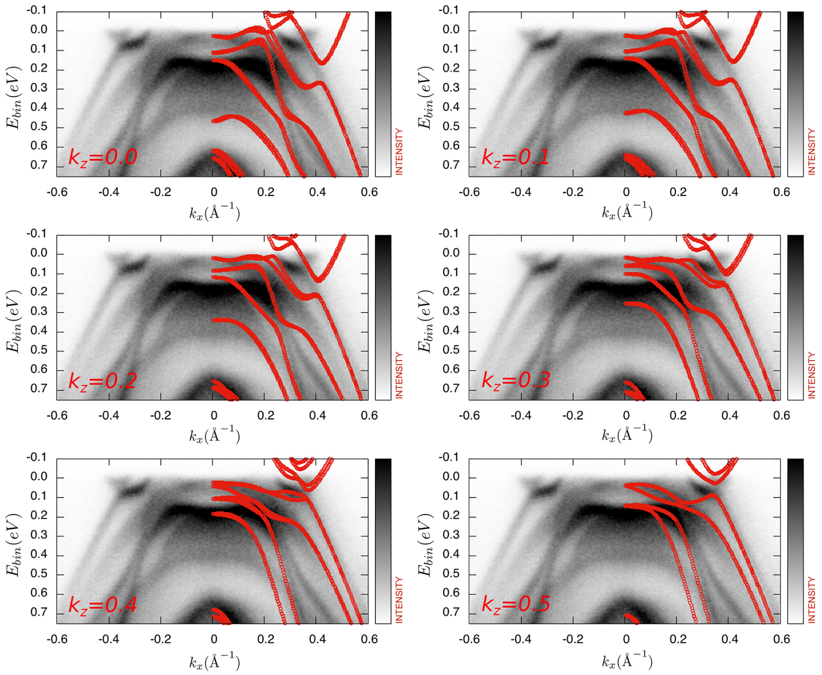

Supplementary Figure 6: Theoretical k_z dispersion. Theoretical DFT bandstructures for bulk WTe2 along the line kx for different out-of-plane k_z momenta overlaid to ARPES spectra. Values reported in the left bottom angle of the panels are in units of $2\pi/c$, and spin-orbit coupling has been taken into account.

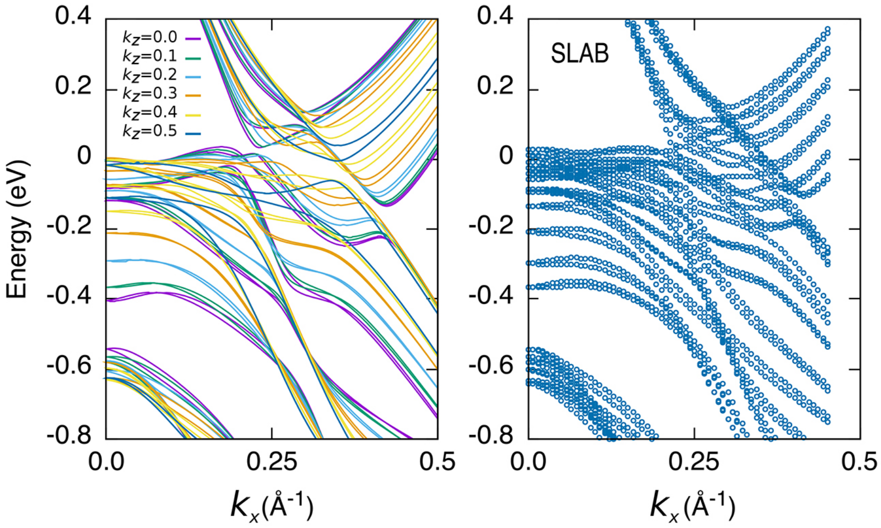

Supplementary Figure 7: bulk k_z dispersion vs. slab bandstructure. Left panel: Theoretical DFT bandstructures for bulk WTe_2 along the line ΓX for different out-of-plane k_z momenta. Values reported in the figure's caption are in units of $2\pi/c$, and spin-orbit coupling has been taken into account. Right panel: DFT bandstructure for a 6-layer-thick slab.

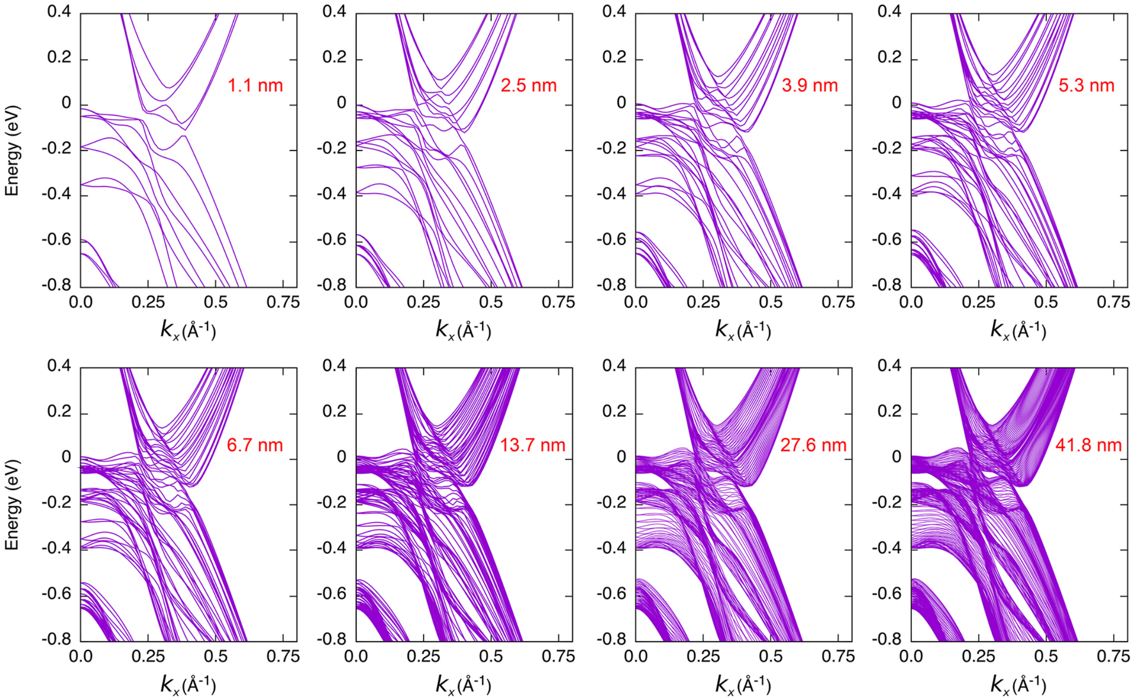

Supplementary Figure 8: Tight binding slab calculations. Ab-initio tight-binding model bandstructures for WTe_2 film thicknesses ranging from 1.1 nm (2 WTe2 layers) to 41.8 nm (60 WTe2 layers) along the ΓX reciprocal direction.

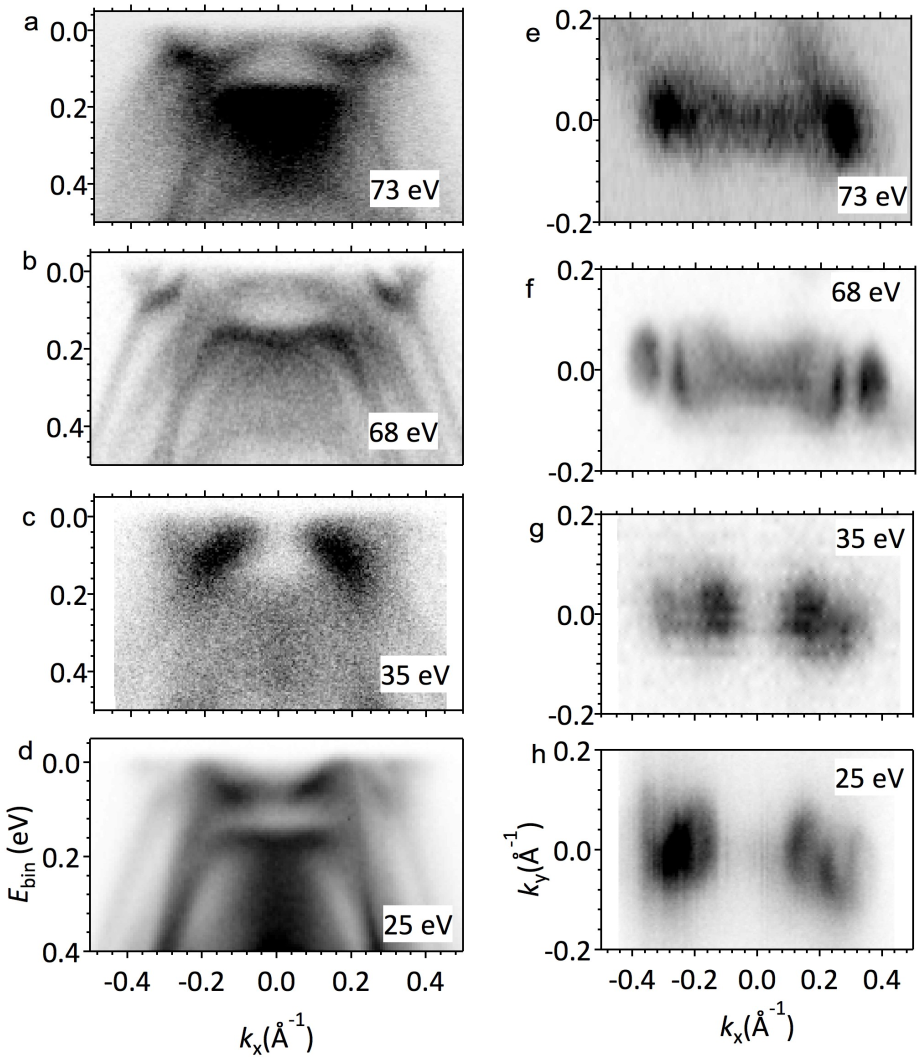

Supplementary Figure 9: Photon energy dependence of band structure. The electronic band structure and Fermi surface are measured by ARPES at various photon energies. Panel a)–d) show the band structure along ΓX measured at photon energy 73, 68, 35, and 25 eV respectively, while panel e)–h) show the respective Fermi surface cuts. A clear photon energy dependence of band structure is observed. However, the overall size of Fermi surface area, and position of electron and hole pockets remain the same for all the energy values used in our experiment. A detailed photon energy dependence study has been done by Pletikosić et al. [7], where they report a detailed k_z dependence.

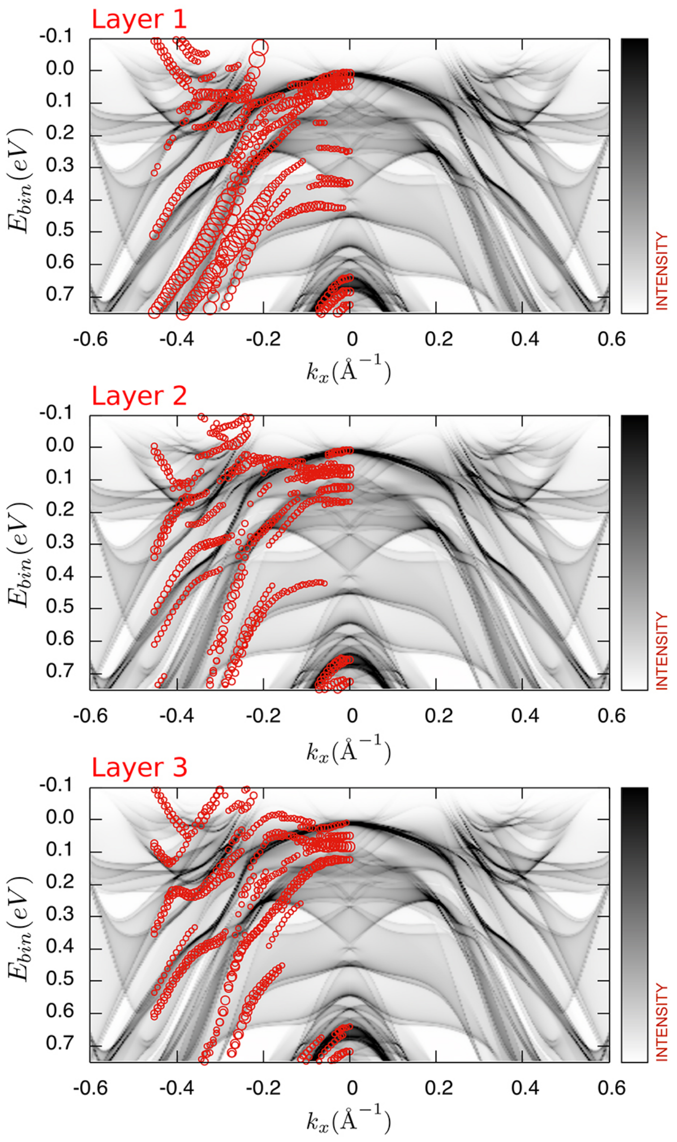

Supplementary Figure 10: Semi infinite system vs slab geometry. Theoretical surface spectral function (Fig. 2f in the main text) overlaid with layered resolved 6-layers-thick slab calculations (Fig. 2c-e).

Supplementary notes

Supplementary Note 1: Inequivalent tellurium atoms at the surface.

The observation of two inequivalent sites for Te in the STM characterization of the surface is confirmed by the core level photoemission results in Supplementary Fig. 1, where Te 4d and W 4f shallow core levels are shown. Unlike the W 4f core level lines, each spin orbit partner of Te 4d is split into two components. The relative intensity of the peaks changes vs. photon energy.

Because the photon energy range is too narrow to allow for cross section effects, this type of variation is the signature of photoelectron diffraction, as already observed in core level of topological insulators [5], and confirming the existence of structurally and chemically inequivalent Te atoms in the Te-W-Te unit at the surface. This inequivalence is further confirmed by our ab-initio-calculated relaxed atomic positions to be present in the bulk and not only on the surface layer. These findings are also consistent with structural data reported in Ref. [6], showing two different W-Te bond lengths (2.71 and 2.81 Å) within each WTe_2 plane.

Supplementary note 2: Surface distortion

Supplementary Fig. 2 shows topographic images at different bias voltages and corresponding Fourier-transform of a representative area of WTe_2. A distortion is clearly visible, as already reported in literature [1, 2]. To assess if the observed distortion is confined at the surface, High Resolution Transmission Electron Microscopy was performed on freshly cleaved crystals at 10.5 K. Supplementary Fig. 3 is a representative Selected Area Electron Diffraction (SAED) taken on a WTe_2 in the [011] zone. Diffraction spots are labeled in the SAED and compatible with those expected for the orthorhombic WTe_2 structure in the [011] zone axis. No signs of sublattices or structural distortions in the bulk of the crystal can be revealed by SAED investigation, confirming previous findings [3].

These results suggest that the observed distortion is confined at the surface region, and that the perturbation induced by the distortion does not give a detectable contribution to electron diffraction in the volume. To further confirm this observation, the difference between diffractograms with and without a distortion have been simulated [4]. Results are shown in Supplementary Fig. 4, where the orthorhombic (left) and 6 degrees distorted (right) simulations are presented: the experimental SAED clearly agrees with the orthorhombic one.

Having ascertained that the distortion is confined at the surface, a further issue is to evaluate if the electronic band structure of the first layer(s) is affected and/or modified, thus possibly influencing the interpretation of the ARPES results. Supplementary Fig. 5 presents band structure calculations with and without the observed surface distortion, calculated for one

monolayer (i.e. in the case of the maximum effect possible), and taking into account the relaxation of neighboring atoms.

The distortion does not significantly modify the overall band structure and, more importantly, the hole-electron compensation is maintained. As for the broken symmetry and possible induced spin-polarization, the produced splitting is of the order of a few meV (< 5 meV) and localized only onto a single monolayer, thus not visible within our experimental resolution.

Supplementary References

1. A. Crossley, S. Myhra, C.J. Sofield: STM analysis of WTe2 surfaces — correlation with crystal and electronic structures - Surface Science **318,** 39-45 (1994)
2. S.W. Hlaa, V. Marinković, A. Prodana, I. Muševiča: STM/AFM investigations of β-MoTe2, α-MoTe2 and WTe2 - Surface Science **352-354**, 105-111 (1996)
3. Ali M. N., Xiong J. et al. Large, non-saturating magnetoresistance in WTe_2, Nature **514**, 205 -208 (2014).
4. P. Stadelmann (2006) JEMS Electron Microscopy Software, JAVA version 4.3030U2015, http://www.jems-saas.ch/home/jemsv4_3030u2015.htm];
5. Kuroda K., Ye. M et al. Experimental verification of the surface termination in the topological insulator $TlBiSe_2$ using core-level photoelectron spectroscopy and scanning tunneling microscopy, Phys. Rev. B **88**, 245308 (2013)
6. Mar A., Jobic S., Ibers A, Metal-metal vs tellurium-tellurium bonding in WTe_2 and its ternary variants $TaIrTe_4$ and $NbIrTe_4$, J. Am. Chem. Soc. **114**, 8963-8971 (1992)
7. Pletikosić I., Ali M. N., Fedorov, A. V. Cava R. J., Valla T., Electronic Structure Basis for the Extraordinary Magnetoresistance in WTe_2, Phys. Rev. Lett. **113**, 216601 (2014)
